# Multi-omics analyses reveal the virulence differentiation underlying natural variation in *Burkholderia gladioli*

**DOI:** 10.1128/aem.01685-25

**Published:** 2025-11-19

**Authors:** Rui Cheng, Tianxing Lv, Pengfei Ji, Bin Ma, Mengcen Wang, Haruna Matsumoto

**Affiliations:** 1 College of Agriculture and Biotechnology, Zhejiang University 162679 , Hangzhou, China; 2 State Key Laboratory of Rice Biology and Breeding, Ministry of Agricultural and Rural Affairs Laboratory of Molecular Biology of Crop Pathogens and Insects, Zhejiang University 12377, Hangzhou, China; 3 Key Laboratory of Biology of Crop Pathogens and Insects of Zhejiang Province, Institute of Pesticide and Environmental Toxicology, Zhejiang University 12377, Hangzhou, China; 4 Department of Chemistry, Zhejiang University 220732, Hangzhou, China; 5 College of Environmental and Resource Sciences, Zhejiang University 366101 , Hangzhou, China; 6 Global Education Program for AgriScience Frontiers, Graduate School of Agriculture, Hokkaido University 527383, Sapporo, Japan; The University of Tennessee Knoxville, Knoxville, Tennessee, USA

**Keywords:** *Burkholderia gladioli*, rice bacterial panicle blight, natural variation, comparative genomics, transcriptomics, virulence differentiation

## Abstract

*Burkholderia gladioli* is a critical pathogen causing bacterial panicle blight in rice, severely threatening global rice yield and grain quality. Here, *B. gladioli* strains were isolated and identified from two rice fields exhibiting markedly different severities of bacterial panicle blight. Although the two strains belong to the same species, they displayed significant differences in phenotype and pathogenicity. Comparative genomic and transcriptomic analyses revealed that natural variation between the strains not only arose from 79 single-nucleotide polymorphisms (SNPs), 12 insertions/deletions (INDELs), and 3 structural variations (SVs) across 27 mutated genes, which may affect protein function and stability, but also coincided with the significant downregulation of genes in multiple virulence-associated pathways, such as two-component systems, bacterial chemotaxis, quorum sensing, and flagellar assembly at the transcriptional level. The combined effects of genetic variation and transcriptional regulation ultimately contributed to the observed differences in pathogenicity. This study uncovers the potential mechanisms by which natural variation in *B. gladioli* influences pathogenicity, providing a theoretical basis and potential molecular targets for the precise control of rice bacterial panicle blight.

**IMPORTANCE:**

This study demonstrates that natural variation in *Burkholderia gladioli*, a major pathogen responsible for bacterial panicle blight in rice, has a significant impact on its pathogenicity and further explores the underlying mechanisms. These findings expand our understanding of how phytopathogens’ virulence differentiates conditions of natural variation, and provide potential molecular targets for the development of novel bactericides. The identification of low-virulence strains and their associated gene variations in this study offers both theoretical and practical foundations for ecological disease management and biocontrol of rice bacterial diseases, highlighting their importance for promoting precision agriculture and sustainable development.

## INTRODUCTION

With the continuous growth of the global population, the demand for staple crops is steadily increasing. As the primary food source for more than half of the world’s population, rice plays a fundamental role in ensuring global food security (1–4). However, rice yield has long been threatened by a variety of diseases, and these threats are expected to intensify under ongoing climate change (5–9). In recent years, members of the genus *Burkholderia* have emerged as major pathogens responsible for bacterial diseases of rice in many regions worldwide, due to their strong infectivity and ability to produce toxins (6, 10–17). Among them, *B. gladioli* is a key pathogen causing bacterial panicle blight of rice. Owing to its high infectivity, prolific reproduction, and strong environmental adaptability, *B. gladioli* poses a persistent threat to rice production globally (18–20).

Currently, chemical control remains the primary method for managing bacterial diseases in rice, including bacterial panicle blight (21). However, the improper use of chemical pesticides can pose risks to crop safety and the surrounding ecosystem. In particular, frequent or excessive application of pesticides may accelerate the development of resistance in plant pathogens, thereby compromising the effectiveness of disease control (22, 23). Although breeding for disease-resistant cultivars represents another major strategy for plant protection, the rapid evolution of plant pathogens often renders these resistant varieties ineffective over time (24). Given the limitations of current management approaches, there is an urgent need to develop more precise and environmentally friendly alternatives to control *B. gladioli*.

The adaptability of plant pathogens determines their ability to infect host plants, survive in diverse environments, and spread across ecological niches. Adaptive shifts across different niches are often associated with variations in phenotypic traits and pathogenicity (25). Studies have shown that prolonged exposure of pathogens to diverse biotic and abiotic selection pressures in natural environments can lead to genomic-level genetic variations, which may alter their virulence (26). For instance, in *Botrytis cinerea*, natural mutations in the *bcvel1* gene disrupt the function of its regulatory protein, thereby impairing fungal development and oxalic acid production, ultimately resulting in significantly reduced virulence (27). Similarly, a 50-year longitudinal study on the New Zealand forest pathogen *Dothistroma septosporum* revealed a substantial decline in virulence over time, attributed to naturally accumulated mutations (28). Although natural variation has been shown to substantially influence the virulence of plant pathogens, such investigations in *B. gladioli* remain limited. In-depth elucidation of the mechanisms underlying natural variation, particularly genetic and transcriptional alterations associated with attenuated virulence, can help identify key pathogenicity factors and provide a theoretical foundation for developing more targeted and environmentally sustainable disease management strategies.

Here, we observed substantial differences in the severity of bacterial panicle blight between two rice field plots. Two bacterial strains were isolated from the panicles collected from each field, and both were identified as *B. gladioli* according to 16S rRNA gene-based phylogenetic analysis despite the fact that they exhibited differences in morphology, phenotypic traits, and virulence in rice. To investigate the molecular basis underlying the natural variation in *B. gladioli*, we conducted comparative genomic and transcriptomic analyses, which revealed potential mechanisms influencing virulence variation. This study provides new insights into the adaptation and evolution of *B. gladioli* and identifies potential molecular targets for the development of more targeted disease management strategies.

## RESULTS

### Naturally variable *B. gladioli* show distinctive phenotypic traits

**Fig 1:**
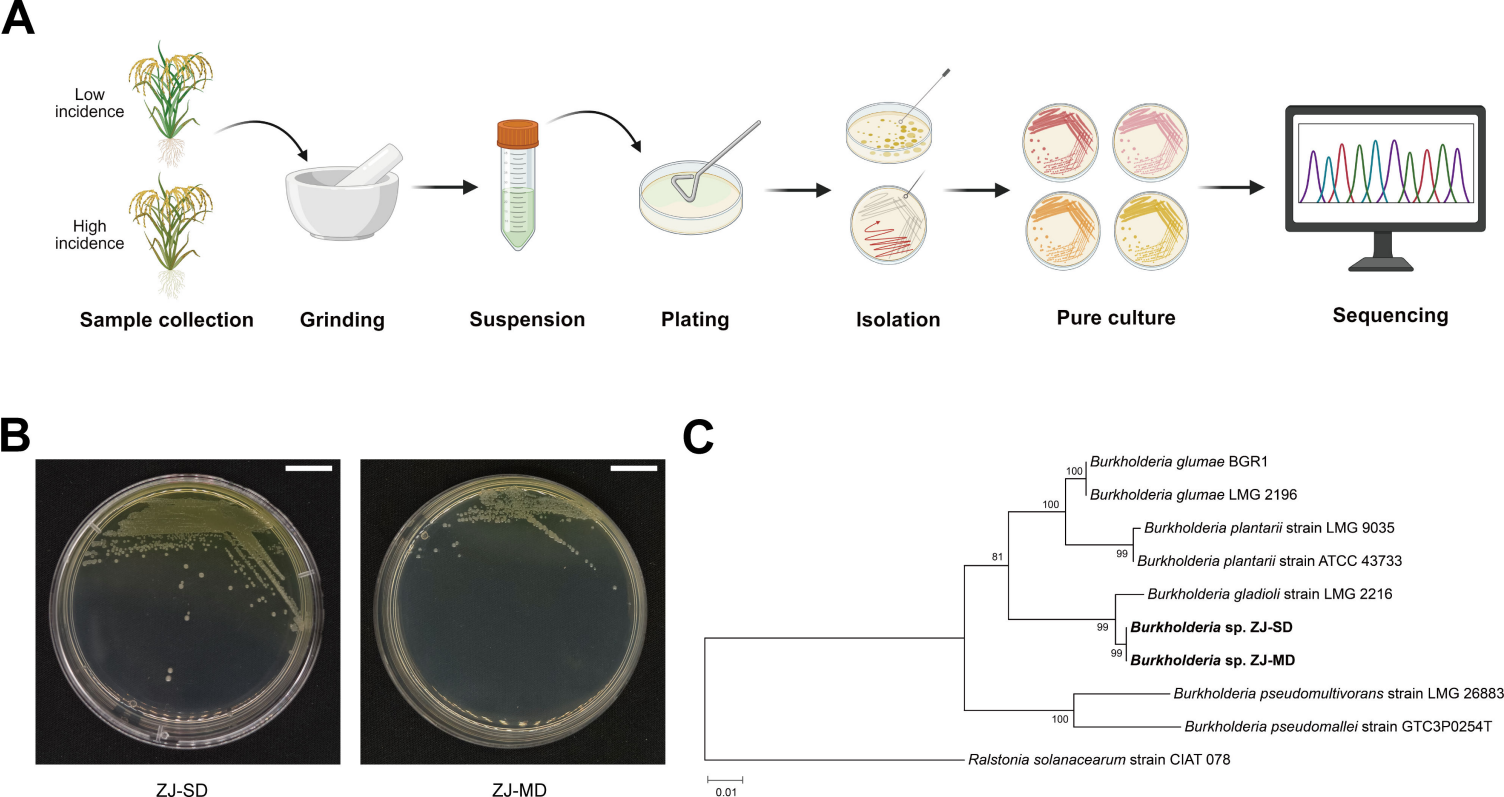
Isolation and identification of *B. gladioli* from rice panicles. (**A**) Schematic workflow of bacterial isolation and identification. Rice panicles were collected from fields with high and low disease incidence. Samples were ground, suspended, plated on LB medium, and single colonies were isolated and purified for sequencing analysis. (**B**) Colony morphology of representative isolates ZJ-SD (severe disease field) and ZJ-MD (mild disease field) grown on LB medium. Scale bar, 1 cm. (**C**) Phylogenetic analysis of the *recA* gene sequences of strains ZJ-SD and ZJ-MD. The phylogenetic tree was constructed based on the maximum likelihood method using the *recA* gene sequences of strains ZJ-SD, ZJ-MD, and other closely-related *B. gladioli* isolates. *Ralstonia solanacearum* was used as an outgroup. The scale indicates 0.01 substitutions per nucleotide position.

We collected diseased panicles from rice fields that exhibited contrasting levels of bacterial panicle blight incidence, which we designated as severe disease field and mild disease field. Subsequent isolation of the pathogens was performed on the rice panicles, and the obtained colonies were identified by 16S rRNA gene sequencing (Fig. 1A). Interestingly, the purified isolates from the same field were highly identical in colony morphology, but the isolates from the different fields showed variations in colony morphology (Fig. 1B) and all isolates were of the same 16S rRNA gene sequences. We, thus, designated the isolate from the severe disease field as strain ZJ-SD and the isolate from the mild disease field as strain ZJ-MD in this study. Notably, ZJ-SD and ZJ-MD shared identical 16S rRNA gene sequences. To further confirm the taxonomic status of ZJ-SD and ZJ-MD, phylogenetic trees based on *recA* and 16S rRNA gene sequences were constructed, which indicated that both strains belonged to *B. gladioli* (Fig. 1C; Fig. S1).

**Fig 2:**
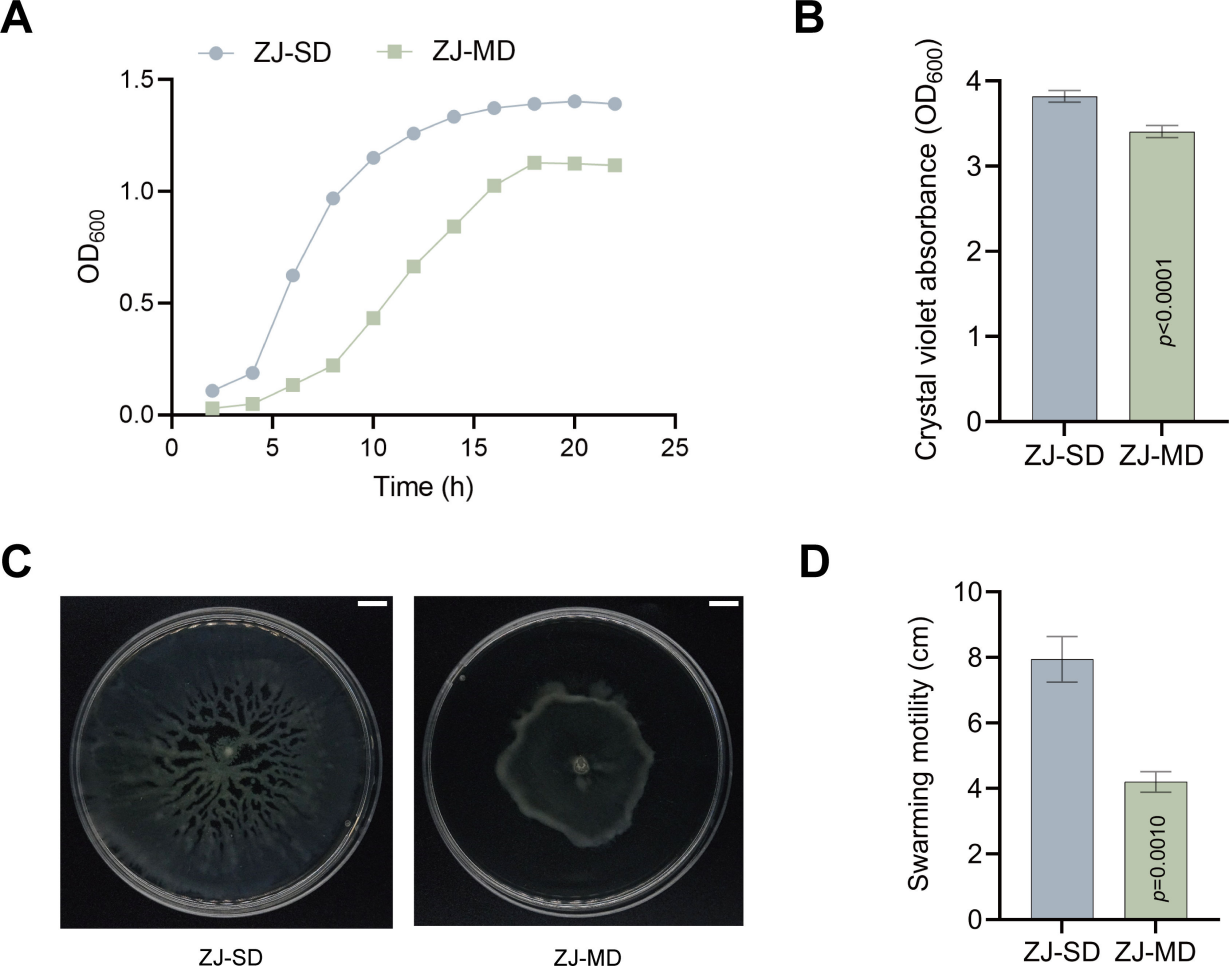
Phenotypic and biological differences between ZJ-SD and ZJ-MD. (**A**) Growth curves of ZJ-SD and ZJ-MD. Values are means ± SD (*n* = 3). (**B**) Quantification of biofilm formation by ZJ-SD and ZJ-MD using crystal violet staining. Values are means ± SD (shown as error bars) (*n* = 3). Statistical significance was determined by two-tailed Student’s-*t* test. (**C**) Representative images of swarming motility of ZJ-SD and ZJ-MD. Scale bar, 1 cm. (**D**) Quantification of swarming motility zone diameter. Values are means ± SD (shown as error bars) (*n* = 3). Statistical significance was determined by two-tailed Student’s-*t* test.

Given the observed differences in field disease severity and colony morphology, we next compared phenotypes of ZJ-SD and ZJ-MD. ZJ-SD formed ivory white, round, smooth, and glossy colonies, while ZJ-MD produced smaller, round colonies with noticeably reduced fullness and gloss (Fig. S2). Additional biological assays demonstrated substantial differences in their growth dynamics: the logarithmic growth phase of ZJ-MD was delayed by approximately 4 hours compared to ZJ-SD, and its stationary-phase OD value was lower (Fig. 2A). Moreover, ZJ-MD exhibited significantly reduced biofilm formation capacity relative to ZJ-SD (Fig. 2B). Swarming motility assays confirmed that ZJ-MD showed significantly impaired motility compared to ZJ-SD (Fig. 2C and D). Despite belonging to the same species, ZJ-SD and ZJ-MD exhibited pronounced differences in colony morphology and other phenotypic traits, prompting further evaluation of their biological properties.

### Pathogenicity divergence between ZJ-SD and ZJ-MD in rice

**Fig 3:**
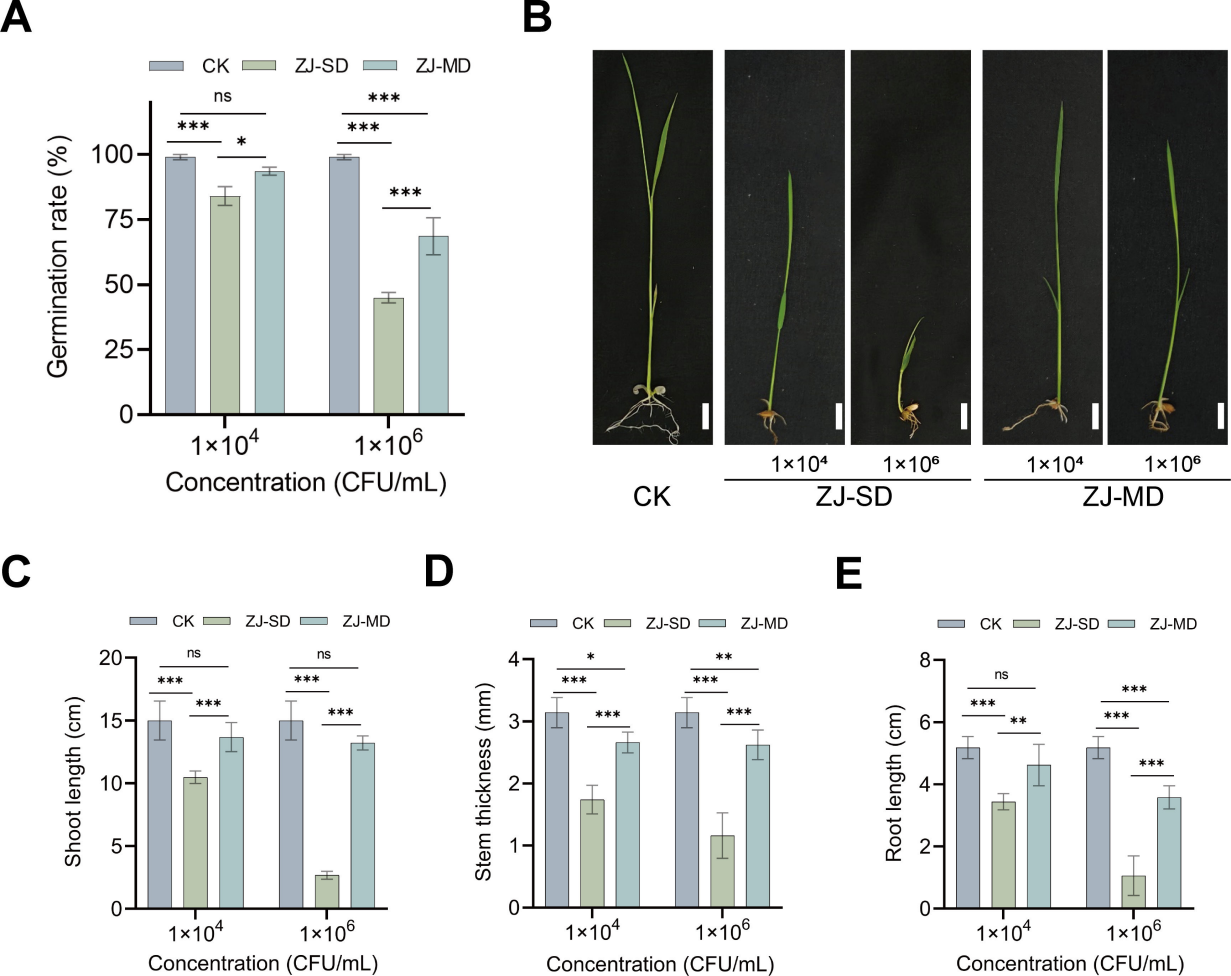
Pathogenic effects of ZJ-SD and ZJ-MD on rice seed germination and seedling growth. (**A**) Germination rate of rice seeds inoculated with different concentrations of ZJ-SD and ZJ-MD (1 × 10^4^ and 1 × 10^6^ CFU/mL). Values are means ± SD (shown as error bars) (*n* = 3). Statistical significance was determined by one-way ANOVA with Tukey’s HSD test. (*P < 0.05, **P < 0.01, ***P < 0.001, ns, not significant). (**B**) Representative images of rice seedlings after seed soaking with different concentrations of ZJ-SD, ZJ-MD, and control (CK) (1 × 10^4^ and 1 × 10^6^ CFU/mL). Scale bars = 1 cm. (**C–E**) Effects of ZJ-SD, ZJ-MD, and control (CK) on rice seedling shoot length (**C**), stem thickness (**D**), and root length (**E**) after seed soaking with different bacterial concentrations (1 × 10^4^ and 1 × 10^6^ CFU/mL). Values are means ± SD (shown as error bars) (*n* = 3). Statistical significance was determined by one-way ANOVA with Tukey’s HSD test. (*P < 0.05, **P < 0.01, ***P < 0.001, ns, not significant).

To further determine whether these phenotypic differences were accompanied by changes in pathogenicity, we compared the virulence of the two strains. Seed soaking assays showed that both ZJ-SD and ZJ-MD inhibited seed germination at a high inoculum concentration (1 × 10^8^ CFU/mL) (Fig. S3). However, at lower concentrations (1 × 10^4^ and 1 × 10^6^ CFU/mL), the germination rate of seeds treated with ZJ-MD was significantly higher than that of the ZJ-SD group (Fig. 3A), and the seedlings in the ZJ-MD group exhibited greater shoot length, stem thickness, and root length compared to those in the ZJ-SD group (Fig. 3B through E).

**Fig 4:**
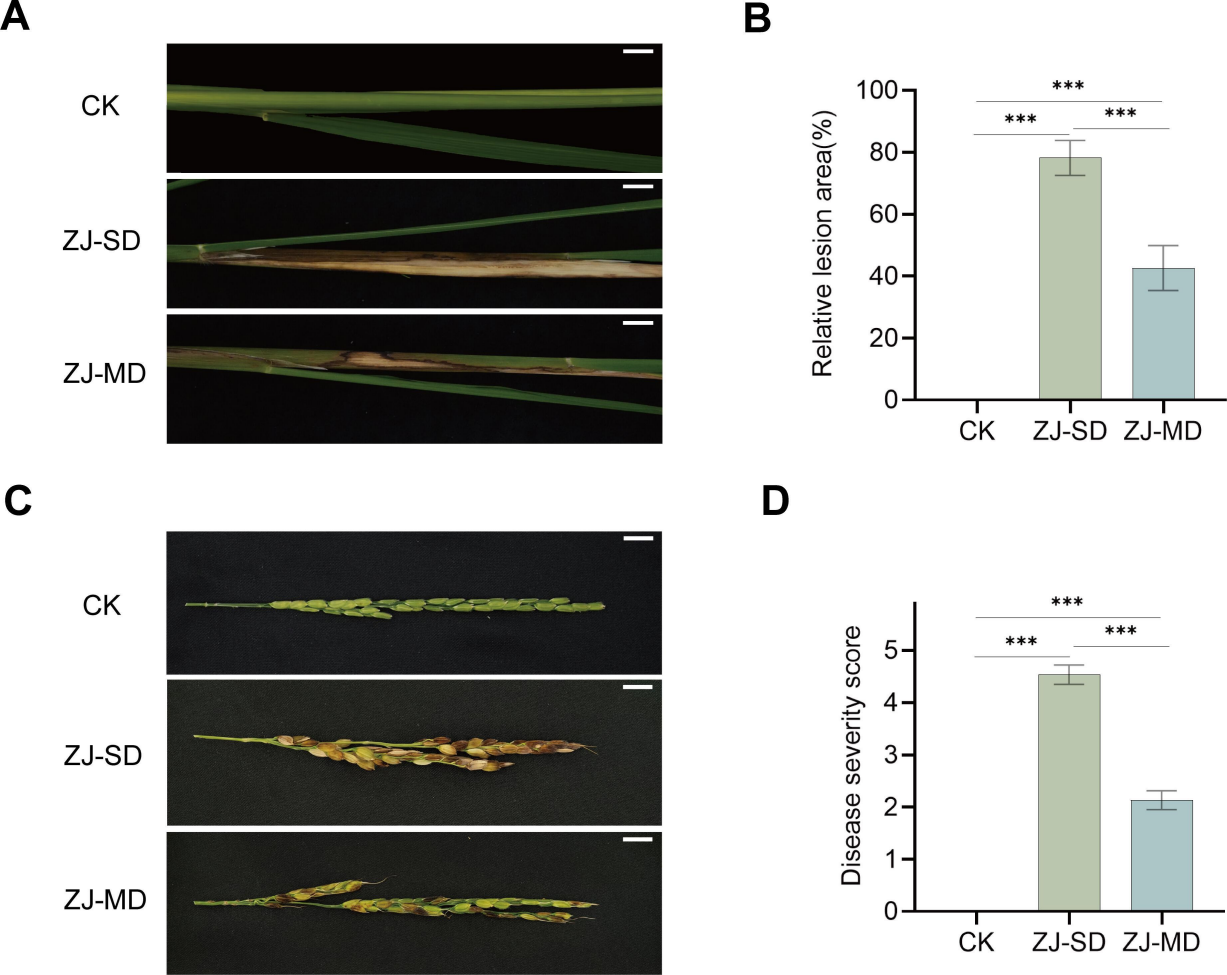
Pathogenic effects of ZJ-SD and ZJ-MD on rice at the booting stage. (**A**) Representative images of rice stems at the booting stage after inoculation with ZJ-SD, ZJ-MD, and control (CK). Scale bar, 1 cm. (**B**) Statistical analysis of relative lesion area in rice stems at the booting stage after inoculation with ZJ-SD, ZJ-MD, and control (CK). Values are means ± SD (shown as error bars) (*n* = 3). Statistical significance was determined by one-way ANOVA with Tukey’s HSD test. (***P < 0.001). (**C**) Representative images of rice panicles after inoculation at the booting stage with ZJ-SD, ZJ-MD, and control (CK). Scale bar, 1 cm. (**D**) Statistical analysis of disease severity scores in rice panicles after inoculation with ZJ-SD, ZJ-MD, and control (CK). Values are means ± SD (shown as error bars) (*n* = 3). Statistical significance was determined by one-way ANOVA with Tukey’s HSD test. (***P < 0.001).

In stem injection assays at the seedling stage, ZJ-MD treatment resulted in much weaker inhibitory effects on rice growth relative to ZJ-SD, especially regarding stem thickness (Fig. S4). When injected at the booting stage, the relative lesion area in rice treated with ZJ-MD was significantly lower than that in the ZJ-SD group (Fig. 4A and B), and the symptoms on panicle grains were markedly alleviated (Fig. 4C and D). Collectively, these results consistently indicate that ZJ-MD exhibits significantly reduced pathogenicity compared to ZJ-SD.

### Modulation of protein function and regulatory networks underlying pathogenicity divergence

**Fig 5:**
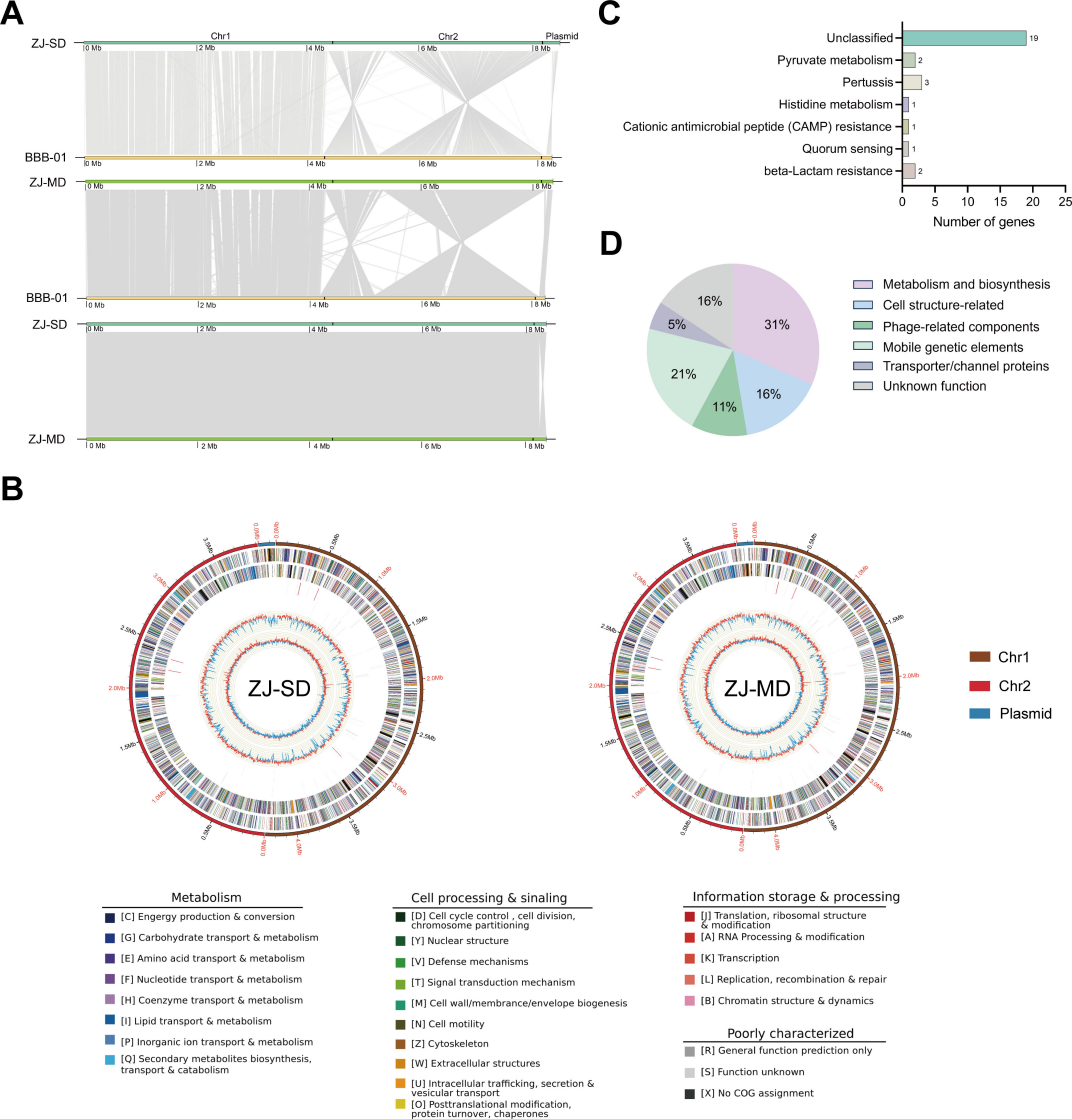
Comparative genomic analysis of ZJ-SD and ZJ-MD. (**A**) Synteny analysis illustrating the conservation of gene order and sequence similarity among strains ZJ-SD, ZJ-MD, and BBB-01. Each axis represents the genomic sequence of one strain, with genes plotted based on their genomic positions. Lines connecting the axes indicate orthologous gene pairs, highlighting regions of high conservation and structural synteny across the genomes. (**B**) Circular genome maps of ZJ-SD and ZJ-MD. Brown, red, and blue rings represent Chr1, Chr2, and the plasmid, respectively. Rings from the outermost to the center represent: (1) scale marks of the genome; (2) protein-coding genes on the forward strand; (3) protein-coding genes on the reverse strand; (4) tRNA (black) and rRNA (red) genes on the forward strand; (5) tRNA (black) and rRNA (red) genes on the reverse strand; (6) GC content; (7) GC skew. Protein-coding genes are color coded according to their COG functional categories. (**C**) KEGG pathway enrichment analysis of the mutated genes. Bars represent enriched pathways, with the number at the end of each bar indicating the number of mutated genes associated with the corresponding pathway. (**D**) Functional classification of the mutated genes based on domain prediction and annotation. The pie chart shows the distribution of the 19 mutated genes across six major functional categories.

To further investigate the genetic basis between ZJ-SD and ZJ-MD, we performed whole-genome sequencing and comparative analysis of the two strains. Synteny analysis of ZJ-SD, ZJ-MD, and the reference strain *B. gladioli* BBB-01 revealed extensive structural conservation, with ZJ-SD and ZJ-MD showing particularly high collinearity (Fig. 5A). Consistently, the average nucleotide identity (ANI) values above 97% between ZJ-MD, ZJ-SD, and the reference *B. gladioli* BBB-01 confirmed that both strains belong to the same species (Fig. S5) (29, 30).

The genome of ZJ-MD consists of two chromosomes and one plasmid, with a total length of 8,357,995 bp and a GC content of 67.98%. A total of 7,213 coding genes were predicted, with coding regions accounting for 86.82% of the genome and an average gene length of 1,005.96 bp. The genome also contains 105 non-coding RNAs, including 15 rRNAs, 66 tRNAs, and 24 sRNAs (Table 1). The genome structure of ZJ-SD is similar to that of ZJ-MD (Fig. 5B). COG functional annotation of protein-coding genes revealed that most genes in both strains were assigned to categories such as transcription, amino acid transport and metabolism, and carbohydrate transport and metabolism. Overall, ZJ-MD and ZJ-SD exhibited highly similar distributions in COG functional categories, indicating consistency in their basic biological functions (Fig. S6).

**TABLE 1:** Comparison of basic genomic features between strain ZJ-MD and ZJ-SD

Feature	ZJ-MD	ZJ-SD
Genome size (bp)	8,357,995	8,358,073
GC content (%)	67.98	67.98
Number of coding genes	7,213	7,220
Total length of coding genes (bp)	7,256,016	7,258,785
Average length of coding genes (bp)	1,005.96	1,005.37
Percentage of genome coding region (%)	86.82	86.85
Number of non-coding RNAs	105	105
Number of rRNAs	15	15
Number of sRNAs	66	66
Number of tRNAs	24	24

Despite the high similarity in genome structure and functional annotation, we performed detailed variation analysis to explore the genetic basis underlying their phenotypic and pathogenic differences, including single nucleotide polymorphisms (SNPs), insertions and deletions (INDELs), and structural variations (SVs). The results showed that ZJ-MD harbored a total of 264 SNPs, 19 INDELs, and 5 SVs compared to ZJ-SD (Fig. S7A). These genomic differences between ZJ-SD and ZJ-MD reflect natural variation within the same species, potentially contributing to the observed phenotypic and pathogenic differences.

The identified SNPs were further classified into non-coding SNPs (ncSNPs), non-synonymous SNPs (nsSNPs), and synonymous SNPs (sSNPs) (Fig. S7B). To pinpoint mutations with potential functional consequences, we focused on variants located within protein-coding regions, including nsSNPs, coding-region INDELs, and SVs. These mutations are more likely to alter protein structure or function and thus influence phenotypic traits or pathogenicity. After filtering, a total of 27 mutated genes were identified across these three categories (Table S1). To investigate the potential biological roles of these genes, we performed KEGG pathway enrichment analysis. Among the 27 genes, 8 genes were mapped to six annotated KEGG pathways: beta-lactam resistance, quorum sensing, cationic antimicrobial peptide (CAMP) resistance, histidine metabolism, pertussis, and pyruvate metabolism (Fig. 5C). These pathways are known to be involved in microbial pathogenicity, stress adaptation, and metabolic regulation, suggesting that the identified mutations may play critical roles in the observed phenotypic and virulence differences between ZJ-SD and ZJ-MD. The remaining 19 mutated genes did not show KEGG pathway annotation and were, therefore, further analyzed through domain-based functional prediction using Pfam (Table 2). Based on domain predictions and functional annotation, the 19 mutated genes were categorized into six major functional groups: metabolism and biosynthesis, cell structure-related proteins, mobile genetic elements, transporter/channel proteins, phage-related components, and proteins of unknown function (Fig. 5D). Genes involved in metabolism and biosynthesis represent the largest proportion, followed by those associated with mobile genetic elements, cell surface structure, and other categories. This functional classification highlights the diverse biological processes potentially affected by natural variation, including metabolic regulation, extracellular matrix formation, genetic mobility, and substance transport. Collectively, these results indicate that natural variation affects a broad spectrum of functional categories, which may, in turn, influence bacterial virulence, environmental adaptation, and physiological characteristics. Such mutations could exert their effects either by directly altering protein function or by modulating broader regulatory networks.

**TABLE 2:** Summary of domain annotation and functional categories of mutated genes

Gene ID	Mutation type	Pfam ID	Variant in domain	Predicted function	Functional category
BJLANFEE_00296	nsSNPs	PF00009, PF00679, PF03144, PF03764, PF14492	Yes	Translational elongation	Metabolism and biosynthesis
BJLANFEE_02717	nsSNPs	PF13649	Yes	Methyltransferase domain	Metabolism and biosynthesis
BJLANFEE_02813	nsSNPs	PF00501, PF00550, PF00668, PF02911, PF13193	Yes	Biosynthetic process, phosphopantetheine binding, catalytic activity	Metabolism and biosynthesis
BJLANFEE_02814	nsSNPs	PF00501, PF00550, PF00668, PF00975, PF13193	Yes	Biosynthetic process, catalytic activity	Metabolism and biosynthesis
BJLANFEE_05428	nsSNPs	PF00501, PF00550, PF00668, PF00975, PF13193	No	Biosynthetic process, catalytic activity	Metabolism and biosynthesis
BJLANFEE_06958	SV	PF01370, PF08338	Yes	Epimerase	Metabolism and biosynthesis
BJLANFEE_01920	nsSNPs	PF13372	Yes	Alginate export	Cell structure-related
BJLANFEE_03943	SV	/^*a*^	/	Pilus assembly protein FimV	Cell structure-related
BJLANFEE_04393	SV	PF01391, PF15984	No	Collagen and collagen-like structural proteins	Cell structure-related
BJLANFEE_02684	nsSNPs	PF09684	No	Phage tail protein	Phage-related components
BJLANFEE_02693	nsSNPs	PF03864	Yes	Phage major capsid protein E	Phage-related components
BJLANFEE_06188	nsSNPs	PF00665	Yes	IS3 family transposase	Mobile genetic elements
BJLANFEE_06199	INDEL	PF01527	No	IS3 family transposase, HTH-Tnp-1	Mobile genetic elements
BJLANFEE_06233	nsSNPs	PF01527	No	DNA transposition, HTH-Tnp-1	Mobile genetic elements
BJLANFEE_06234	nsSNPs	PF13276, PF13683	No	Integrase core domain protein, HTH-21	Mobile genetic elements
BJLANFEE_07078	nsSNPs	PF07690	Yes	Major facilitator superfamily	Transporter/channel proteins
BJLANFEE_03143	nsSNPs	PF06073	Yes	Unknown function	Unknown function
BJLANFEE_04146	nsSNPs	/	/	Unknown function	Unknown function
BJLANFEE_07080	nsSNPs	/	/	Unknown function	Unknown function

^*a*^ / indicates that the Pfam ID could not be predicted, and it reflects the uncertainty regarding the presence of mutations within the domain.

### Transcriptomic reprogramming drives attenuated virulence in ZJ-MD

**Fig 6:**
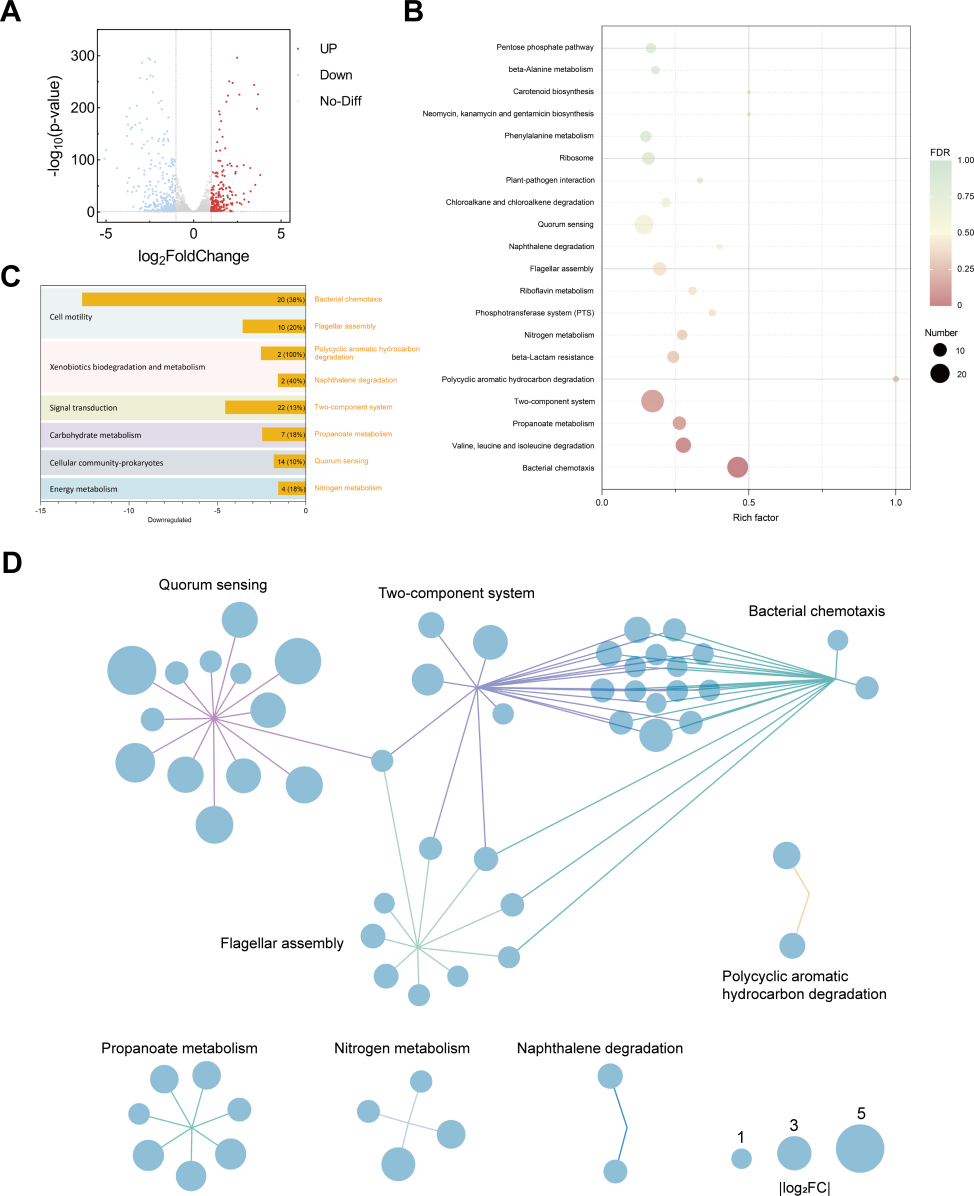
Transcriptomic profiling and pathway enrichment analysis of ZJ-SD and ZJ-MD. (**A**) Volcano plot showing differentially expressed genes (DEGs) in ZJ-MD relative to ZJ-SD. Red dots represent significantly upregulated genes, blue dots indicate significantly downregulated genes, and gray dots indicate genes with no statistically significant change (|log_2_FoldChange| > 1, *P* < 0.05). (**B**) KEGG pathway enrichment analysis of differentially expressed genes (DEGs). The top 20 significantly enriched KEGG pathways are shown. Rich factor represents the ratio of the number of DEGs annotated to a given pathway to the total number of genes annotated to that pathway. The color of each dot corresponds to the false discovery rate (FDR). The size of each dot represents the number of DEGs enriched in the corresponding pathway. Pathways are ranked by FDR. (**C**) Enriched KEGG pathways with significantly down-regulated genes. The *x*-axis indicates *P* values (the distance is log10-transformed) of KEGG pathway enrichments. The numbers in the plot show the number of DEGs in each enriched pathway, with the percentage (in parentheses) of all genes in the corresponding pathways. The enriched pathways with significantly downregulated genes were further clustered in six high-level pathways/processes, which are indicated by different background colors. (**D**) Network analysis of the relationship between significantly downregulated genes and the corresponding enriched KEGG pathways. Each gene is indicated with a blue circle and the relative fold-change (log2-transformed) of gene expression is indicated by circle size. Different pathways of these genes are indicated with lines in different colors. Colored lines link genes to enriched KEGG pathways, and multiple lines indicate that a gene maps to more than one pathway.

Given the multiple genetic variations identified between ZJ-MD and ZJ-SD in comparative genomic analysis, including mutations in genes associated with transcriptional regulation and regulatory network components, we next sought to determine whether these differences resulted in changes at the broader transcriptomic level. The transcriptome results identified a total of 594 significantly differentially expressed genes (DEGs) in ZJ-MD relative to ZJ-SD, of which 250 were upregulated and 344 were downregulated (Fig. 6A; Table S2). KEGG pathway enrichment analysis of all DEGs revealed 91 enriched pathways. The top 20 significantly enriched pathways encompass a variety of biological functions, including cell motility, signal transduction, drug resistance, and various metabolic processes (Fig. 6B; Fig. S8). The large number and diversity of affected pathways underscore a broad transcriptional shift at the network level.

Downregulated genes accounted for the majority of DEGs and were significantly enriched in pathways associated with pathogenicity. Therefore, we focused on KEGG pathways associated with these downregulated genes. In-depth analysis of the top eight significantly enriched KEGG pathways for downregulated genes revealed that they clustered into six functional categories: cell motility, xenobiotics biodegradation and metabolism, signal transduction, carbohydrate metabolism, prokaryotic cellular community, and energy metabolism (Fig. 6C). Downregulation of these pathways likely impairs multiple physiological functions in ZJ-MD, thereby contributing to its reduced pathogenicity.

To further investigate the interactions among these significantly downregulated pathways, we conducted a network analysis of the genes involved in these eight KEGG pathways. The results showed that the two-component system and bacterial chemotaxis served as major network nodes connected with the quorum sensing and flagellar assembly pathways, forming a tightly integrated functional module, suggesting that these downregulated pathways cooperatively participate in the regulation of ZJ-MD virulence (Fig. 6D). Taken together, the downregulation of the two-component system, bacterial chemotaxis, quorum sensing, and flagellar assembly in ZJ-MD points to a coordinated weakening of environmental sensing, signal relay, population coordination, and motility. This coordinated suppression provides a mechanistic basis for the attenuated phenotypes and reduced pathogenicity of ZJ-MD relative to ZJ-SD.

## DISCUSSION

Taxonomic analysis revealed that ZJ-SD and ZJ-MD belong to the same species, yet they exhibited marked differences in both phenotype and pathogenicity. This finding suggests that, even within the same geographic area and under similar ecological conditions, conspecific phytopathogens may accumulate genetic variation over time as a result of prolonged exposure to diverse natural selection pressures, such as host resistance, antagonistic interactions within the field microbiome, fluctuations in climatic conditions, and agricultural management practices, ultimately driving divergence in their phenotypic traits and pathogenic potential (31–34). Such natural variation may profoundly impact the outbreak dynamics, transmission risk, and long-term persistence of field diseases and also poses greater challenges for the selection of resistant cultivars and the implementation of precision field management strategies (35, 36).

Comparative genomic analysis showed that ZJ-SD encodes a small number of additional genes compared with ZJ-MD (Table 1). Most of these are annotated as hypothetical proteins or proteins of unknown function, and their biological roles remain unclear, warranting further investigation to determine whether they may contribute to the strain divergence. In addition, SNP, INDEL, and SV detection ultimately identified 27 mutated genes (Table S1). KEGG pathway enrichment analysis revealed that these genes are primarily involved in key biological pathways related to microbial adaptation, pathogenicity regulation, and metabolism (Fig. 5C). Further domain-based functional predictions indicated that these genes participate in a range of biological processes, including metabolic regulation, signal transduction, and substrate transport (Table 2). For example, BJLANFEE_01920 encodes an alginate export protein, which may contribute to cell surface architecture and biofilm formation—processes that have been demonstrated to play crucial roles in bacterial colonization, stress tolerance, and virulence (37, 38). Several genes, such as BJLANFEE_02813 and BJLANFEE_05428, contain domains associated with biosynthetic and catalytic processes. Although such functions are widely present in cellular metabolism, these domains may also influence the synthesis or modification of metabolites that potentially contribute to virulence (39, 40). Notably, the set of mutated genes includes not only IS3 family transposases but also proteins containing HTH (helix-turn-helix) domains and key factors involved in quorum sensing pathways. The activity of IS family transposases may impact bacterial adaptability and pathogenicity by mediating genome rearrangements or regulating the expression of virulence-associated genes (41–43). HTH domains are widely found in transcription factors and confer DNA-binding and gene regulatory functions, indicating that these mutations could directly affect transcriptional regulation (44, 45). Among these, BJLANFEE_06188 carried mutations within the IS3 transposase domain, while other mutations were located outside annotated domains (Table 2). Quorum sensing, as a central bacterial signaling system, modulates motility, biofilm formation, and the expression of multiple virulence factors, thereby orchestrating multilayered transcriptional networks that regulate pathogenic phenotypes (46, 47). It is also worth noting that several of these mutated genes remain functionally uncharacterized. Although their specific biological roles await further elucidation, their presence suggests the existence of previously unrecognized mechanisms within the adaptive regulatory networks of *B. gladioli*, warranting additional investigation.

Transcriptomic analysis revealed that there were no significant differences in the expression of these 27 mutated genes. This observation suggests that the phenotypic effects of these mutations may not be mediated by direct alterations in their own transcription but are more likely to arise from changes at the levels of protein function, structural stability, or regulatory activity. For instance, domain alterations or functional loss in some genes could affect the DNA-binding affinity or signal-sensing capacity of their protein products, thereby influencing downstream signal transduction and transcriptional regulatory networks (48, 49). Further KEGG enrichment analysis showed that, in ZJ-MD, several pathways associated with bacterial motility, signal transduction, energy metabolism, and community behaviors were significantly downregulated, particularly those related to two-component systems, bacterial chemotaxis, quorum sensing, and flagellar assembly (Fig. 6C and D). The downregulation of these pathways directly impaired ZJ-MD’s biofilm formation, motility, and environmental adaptability, ultimately resulting in reduced pathogenicity, a finding that was highly consistent with our phenotypic and pathogenicity assays. In addition to the downregulated virulence-associated pathways, ZJ-MD also exhibited a number of upregulated genes, which may represent strain-specific adaptations or general stress responses. The precise roles of these genes remain unclear and warrant further investigation in future studies. Taken together, comparative genomic and transcriptomic analyses indicate that natural variations and the associated transcriptional changes in field isolates collectively contribute to the observed differences in phenotype and virulence.

Within *B. gladioli*, similar patterns have also been observed among different field isolates. Previous studies have revealed diversity in the AHL-type QS system of this species, with compositional variations capable of markedly reprogramming the regulatory network (50). In *B. glumae*, the transcription factor QsmR has been identified as a central QS regulator that directly controls motility, biofilm formation, and virulence (51). These findings suggest that QS serves as an integrative hub coordinating multiple virulence-related pathways. The coordinated downregulation of such pathways observed in ZJ-MD likely reflects the weakening of a similar regulatory node, thereby leading to an attenuation of virulence. Chemotaxis and motility are widely recognized as essential traits for host colonization in phytopathogenic bacteria (52), and recent evidence further indicates that plant-derived acetylcholine signals can drive pathogen chemotaxis and thereby influence infection opportunities (53). Thus, the significant downregulation of pathways related to chemotaxis and motility in ZJ-MD suggests that its capacity for host recognition and initial invasion is severely compromised, providing a reasonable explanation for its attenuated virulence in the field. In addition, studies have shown that QS and DNA methylation exert opposing roles in regulating bacterial phase variation, enabling transcriptional reprogramming without altering the expression of the mutated genes themselves (54). This mechanism is consistent with our observation that mutations did not change their own expression levels but were accompanied by the downregulation of downstream regulatory networks. Collectively, these insights provide an explanation for the transcriptional suppression and reduced pathogenicity observed in ZJ-MD.

The natural variation in *B. gladioli* identified in this study not only resulted in differences in phenotype and pathogenicity but also suggested that such mutations may have complex ecological consequences under natural conditions. On the one hand, certain mutations may reduce virulence or modulate quorum-sensing behaviors, thereby enhancing the ability of the pathogen to survive and persist in the host or environment, which could provide a selective advantage for long-term colonization and transmission (55). On the other hand, these variations may attenuate pathogenicity, potentially placing the pathogen at a competitive disadvantage when interacting with other microbes in the ecological niche (56).

This work focused on two field isolates that exhibited markedly different levels of pathogenicity. While this design highlights the association between natural variation and virulence divergence, its representativeness needs to be validated across a broader collection of isolates. Comparative genomic and transcriptomic analyses revealed potential links between natural variation and downstream regulatory networks, but the precise molecular targets remain incompletely defined. Future studies should incorporate functional assays, such as gene complementation and protein-protein interaction analyses, to directly elucidate the detailed regulatory mechanisms. These further investigations may provide a more comprehensive understanding of how natural variation shapes regulatory networks and virulence, thereby offering valuable insights for the development of more targeted strategies for disease management.

In summary, our findings show that ZJ-MD carries natural variation relative to ZJ-SD, accompanied by coordinated transcriptional suppression of pathways involved in signaling, motility, and collective behaviors. These molecular changes align with the observed phenotypic alterations, including reduced biofilm formation, impaired motility, and slower growth dynamics, which collectively explain the reduced virulence of ZJ-MD in rice. The downregulation of key regulatory networks and virulence-related pathways, such as quorum sensing and motility, provides valuable insights into the mechanisms driving pathogenicity differences. These results not only enhance our understanding of the genetic basis of virulence but also offer new perspectives on the diversity, adaptation, and evolution of plant pathogens. The key mutated genes and regulatory networks identified in this study represent promising molecular targets for disease management. For example, these genes may serve as molecular markers to facilitate the selection of high- or low-virulence strains, thereby improving the accuracy of field pathogen monitoring and epidemic risk assessment. In addition, the identification and application of naturally attenuated strains provide a theoretical basis for developing biocontrol strategies based on antagonistic microbes, which hold promise for reducing reliance on chemical pesticides and achieving greener, more sustainable agricultural disease management.

## MATERIALS AND METHODS

### Isolation and identification of panicle bacteria

Rice panicles exhibiting typical symptoms of bacterial panicle blight were collected in 2022 from two rice fields with markedly different disease severities in Xiaoshan District, Hangzhou, Zhejiang Province, China (30.18°N, 120.26°E). Field disease severity of bacterial panicle blight was scored on a 0-9 ordinal scale, where 0 represented no symptoms and 9 indicated the most severe panicle damage (57). The average field score was 7.5 in the severe-disease field and 2.0 in the mild-disease field. To obtain bacterial isolates, rice panicles were surface-rinsed with 75% ethanol solution (prepared with absolute ethanol and sterile water) for 60 s, washed three times with sterile water, and dried with sterilized paper. Symptomatic grains were cut and ground in a sterile mortar with 2 mL sterile water, and the resulting suspension was serially diluted 10- and 100-fold. Then, 100 µL of each dilution was spread onto LB agar plate (BD) and incubated at 28°C for 3 days. Well-separated single colonies were picked and streaked onto fresh LB agar plate until pure cultures were obtained. Genomic DNA was extracted from purified isolates using a CTAB-based DNA extraction kit (Acmec, China), and the 16S rRNA gene was amplified and sequenced for bacterial identification. For the *Burkholderia* genus, specific primers based on the marker gene *recA* (58) were additionally used to identify them at species level. Each primer pair for PCR-amplification of 16S rRNA and *recA* in *B. gladioli* was then designed using Primer-BLAST (NCBI) as: 16S rRNA forward 5’- TTCCGGTTGATCCTGCCGGA-3’ and 16S rRNA reverse 5’- AAGGAGGTGWTCCARCC -3’; *recA* forward 5’- ATGGAAGATAGCAAGAAAGGCGC-3’; and *recA* reverse 5’- TCACTCTTCTTCGTCCAGGATCTC-3’. The schematic workflow of bacterial isolation and identification was created with BioRender.com.

### Measurement of bacterial growth curves

Overnight cultures of strains ZJ-SD and ZJ-MD were grown in LB broth at 28°C with shaking at 200 rpm. Cells were harvested by centrifugation at 5,000 × *g* for 5 min and resuspended in fresh LB broth, and the cell density was adjusted to an OD_600_ of 0.6-0.8 as measured with a microplate reader (TECAN, Switzerland). The cultures were then diluted 1:100 with fresh LB broth, and 200 µL aliquots were dispensed into each well of a 96-well microplate. The plate was incubated at 28°C with shaking at 200 rpm, and OD_600_ was measured every 2 h for 22 h. Growth curves were generated based on the collected data.

### Biofilm quantification assay

ZJ-SD and ZJ-MD were cultured in LB broth at 28°C with shaking at 200 rpm until mid-log phase. Two hundred microliters of the bacterial suspension was added to three replicate wells of a 96-well plate. Plates were incubated statically at 28°C for 48 h to allow biofilm formation. Quantification of biofilm biomass was performed using the crystal violet staining method as previously described (15).

### Swarming motility assay

ZJ-SD and ZJ-MD were grown overnight in LB medium. The cultures were then adjusted to an OD_600_ of 0.6. A 5 µL aliquot of the standardized bacterial suspension was spotted onto the center of a semi-solid LB agar plates (0.5%, wt/vol agar). Plates were incubated at 28°C for 24-48 h. The diameter of the motility zone was measured to quantify bacterial motility.

### Seed soaking assay for evaluation of disease resistance

From each treatment group, 100 rice seeds were randomly sampled and divided into ten batches (ten seeds per batch). All seeds were surface-sterilized as previously reported (59). To confirm the absence of epiphytic microorganisms, the sterilized seeds were placed on ten LB agar plates (ten seeds per plate) and incubated at 28°C for 48 h; the absence of visible colonies confirmed complete sterilization. Inoculum concentrations of 1 × 10⁶ and 1 × 10^8^ CFU/mL represent mid/high levels commonly used in rice–*Burkholderia* pathogenicity assays (60), and 1 × 10^4^ CFU/mL was included to examine responses under low inoculum conditions. The epiphyte-free seeds were then soaked in 10 mL of bacterial suspension (either ZJ-SD or ZJ-MD, at 1 × 10^4^, 1 × 10⁶, or 1 × 10^8^ CFU/mL) in a 9 cm diameter Petri dish at 28°C until germination. Ten seeds at the early germination stage were randomly collected and transferred into 10 cm high glass dishes containing 25 mL of half-strength Murashige and Skoog (MS) medium solidified with 0.3% gellan gum. The seedlings were incubated in a growth chamber at 28°C, 80% relative humidity, and a 12 h light/12 h dark photoperiod. Stem and root growth were monitored at 5 days and measured after 10 days of incubation. Differences in seedling growth and disease resistance were evaluated among the treatment groups.

### Stem injection assay at seedling and booting stages

ZJ-SD and ZJ-MD were cultured in LB broth at 28°C with shaking until mid-log phase and then diluted to the desired concentrations (1 × 10^4^, 1 × 10⁶, and 1 × 10^8^ CFU/mL for seedling stage; 1 × 10⁶ CFU/mL for booting stage). For the seedling stage, 1 mL of each bacterial suspension was injected into the stem base of rice seedlings using a sterile disposable syringe, with sterile water as the negative control. For the booting stage, 1 × 10⁶ CFU/mL was adopted as a single standardized inoculum, providing a robust balance between consistent disease expression and panicle integrity. The same procedure was applied, and 1 mL of bacterial suspension was injected from the base to the top of the booting panicle until overflow, with sterile water as the control. After 7 days, plant growth parameters and disease symptoms were recorded. The disease relative lesion area was calculated using the Otsu method as previously described (61). The disease severity of bacterial panicle blight was assessed using a previously described rating scale (62).

### Genome sequencing, assembly, and annotation

Genomic DNA of strains ZJ-SD and ZJ-MD was extracted and sequenced using both the Illumina NovaSeq 6000 platform (Illumina, USA) with a 350 bp insert size and the Oxford Nanopore PromethION platform (Oxford Nanopore Technologies, UK) following the manufacturer’s protocols. Illumina paired-end libraries were constructed using the NEBNext Ultra II DNA Library Prep Kit (NEB, USA), and Nanopore sequencing libraries were prepared using the Ligation Sequencing Kit SQK-LSK109 (Oxford Nanopore Technologies, UK). Raw reads from Illumina were filtered and quality-checked with Trimmomatic v0.39 and FastQC v0.11.9. Nanopore reads were quality-controlled using Nanoplot v1.20.0. Low-quality reads (Phred score <20) and reads shorter than 1,000 bp were removed.

Hybrid *de novo* assembly was performed using Unicycler v0.4.8 (https://github.com/rrwick/Unicycler) to combine Illumina and Nanopore reads. The assembly was polished using Pilon v1.24 (https://github.com/broadinstitute/pilon) and evaluated with QUAST v5.0.2. Gene prediction was performed using Prodigal v2.6.3. tRNA and rRNA genes were identified by tRNAscan-SE v1.4 and RNAmmer v1.2, and other ncRNAs were annotated by comparison with the Rfam v14.1 database. Repetitive sequences were detected with RepeatMasker v4.1.2-p1 (http://www.repeatmasker.org/) and Tandem Repeats Finder v4.09. Prophages and genomic islands were identified by PHASTER (http://phaster.ca/) and IslandPath-DIMOB v1.0.0. CRISPR loci were predicted with CRISPRdigger v1.0.

Functional annotation of coding sequences (CDSs) was performed by BLASTp (v2.12.0+) and Diamond v2.0.14 against the NCBI NR, Swiss-Prot, COG, KEGG, GO, Pfam, CAZy, PHI, VFDB, and CARD databases (parameters: *e*-value ≤1*e*-6). Signal peptides and transmembrane domains were predicted by SignalP v5.0, LipoP v1.0, and TMHMM v2.0. Subcellular localization was predicted using PSORTb v3.0. Comparative genomic analyses, including ANI, were performed using FastANI v1.33. The genome map was visualized with Circos v0.69-9. All analyses were performed using default parameters unless otherwise stated.

### Comparative genomic analysis

The ANI between genomes was calculated using FastANI (https://github.com/ParBLiSS/FastANI). Genome synteny analysis was conducted to evaluate the collinearity of homologous genes between genomes, with results visualized using the output from FastANI. For identification of genomic variations, including single nucleotide polymorphisms (SNPs), insertions and deletions (INDELs), and structural variations (SVs), the two complete genome sequences (including plasmid sequences) were aligned using MUMmer v4 (https://github.com/mummer4/mummer). SNP, INDEL, and SV events were called and annotated with the reference GFF files. Detailed variation reports were generated, and only high-confidence variants were considered for downstream analysis. All analyses were performed using default parameters unless otherwise specified.

### Transcriptome profiling of ZJ-SD and ZJ-MD

ZJ-SD and ZJ-MD were grown in LB broth at 28°C, 200 rpm to mid-log phase and harvested by centrifugation; ≥200 mg wet biomass per replicate (*n* = 3) was collected. Total RNA was extracted from ZJ-SD and ZJ-MD cultured under identical conditions using TRIzol reagent (Invitrogen, USA). RNA quality and integrity were assessed with an Agilent 2100 Bioanalyzer (Agilent Technologies, USA). RNA-seq libraries were prepared with an insert size of 380 bp and sequenced on the Illumina platform (paired-end, 2 × 150 bp) by Personalbio (Shanghai, China). Raw reads in FASTQ format were filtered using Cutadapt (v1.18) to remove adapters and low-quality reads (average Q-score <20). Clean reads were aligned to the *B. gladioli* reference genome (GCF_016698705.1) using Bowtie2 (v2.3.5). Gene expression levels were calculated as FPKM using HTSeq (v0.6.1p2). Differentially expressed genes (DEGs) were identified using DESeq (|log_2_ fold change| > 1 and *P*-value < 0.05). KEGG pathway enrichment of DEGs was performed with clusterProfiler (hypergeometric ORA) using the KEGG database (http://www.kegg.jp/kegg/); all detected genes served as the background and pathways with *P* < 0.05 were considered enriched. KEGG network visualization and gene co-expression network analysis were performed using Cytoscape v3.8.0. The identified DEGs are listed in Table S2.

### Statistical analyses

All data are presented as mean ± standard deviation (SD) of at least three independent biological replicates. Statistical significance was determined using one-way analysis of variance (ANOVA) and Student’s-*t* test (*P* < 0.05) in SPSS v26.0. All graphs, including bar charts and line graphs, were generated using GraphPad Prism 9.

## Data Availability

All raw sequence data have been deposited in the Sequence Read Archive of NCBI. ZJ-SD and ZJ-MD genomes were deposited under BioProject accessions PRJNA1304885 and PRJNA1304894, respectively. Transcriptome data sets were deposited under accession PRJNA1303810.

## SUPPLEMENTAL MATERIAL

Supplemental figures aem.01685-25-s0001.pdf Figures S1 to S8.

Table S1 aem.01685-25-s0002.docx Summary of 27 mutated genes and counts of SNPs, INDELs, and SVs.

Table S2 aem.01685-25-s0003.xlsx DEGs identified in ZJ-MD relative to ZJ-SD.

ASM does not own the copyrights to Supplemental Material that may be linked to, or accessed through, an article. The authors have granted ASM a non-exclusive, world-wide license to publish the Supplemental Material files. Please contact the corresponding author directly for reuse.
