# Supplementary material for: Multi-omics analyses reveal the virulence differentiation underlying natural variation in Burkholderia gladioli
Source: Appl Environ Microbiol. 2025 Nov 19;91(12):e01685-25. doi: 10.1128/aem.01685-25 (PMC12724388; doi:10.1128/aem.01685-25)
Supplement: Supplemental figures — Figures S1 to S8. [file aem.01685-25-s0001.pdf]

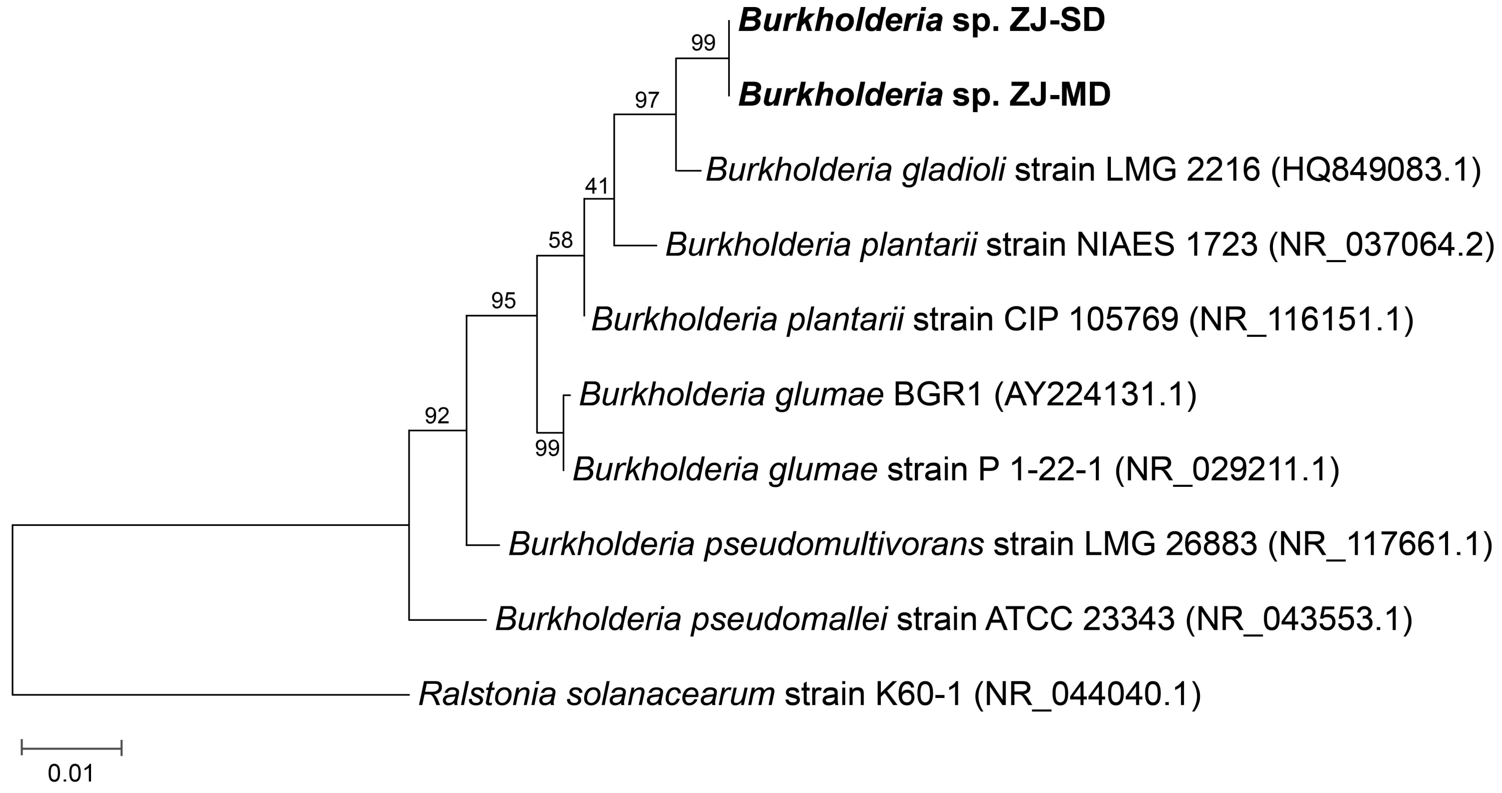

Fig. S1 Phylogenetic analysis of the 16S rRNA gene sequences of strains ZJ-SD and ZJ-MD. The phylogenetic tree was constructed based on the maximum likelihood method using the 16S rRNA gene sequences of strains ZJ-SD, ZJ-MD, and other closely-related *B. gladioli* isolates. *Ralstonia solanacearum* was used as an outgroup. The scale indicates 0.01 substitutions pernucleotide position.

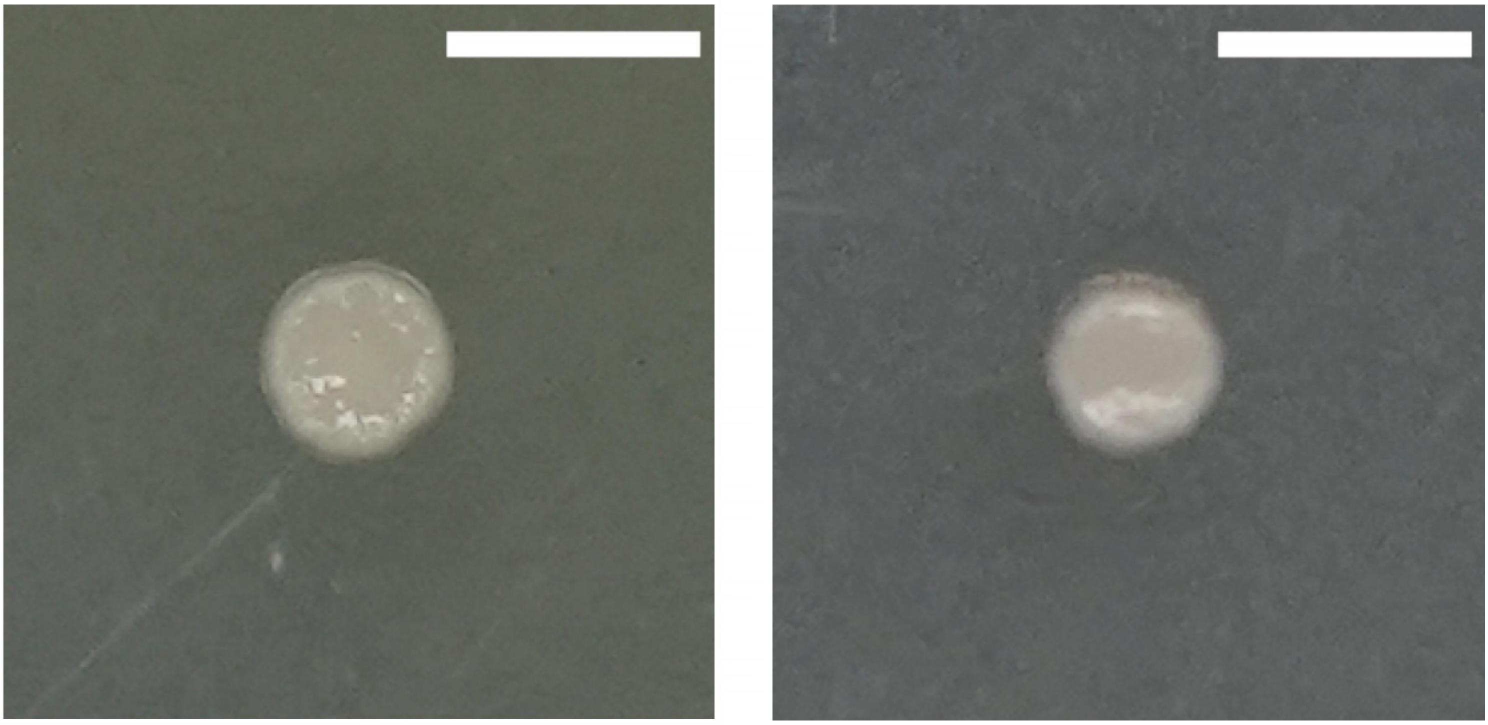

ZJ-SD ZJ-MD

Fig. S2 Representative colonies of ZJ-SD and ZJ-MD on LB medium. Scale bar, 2 mm.

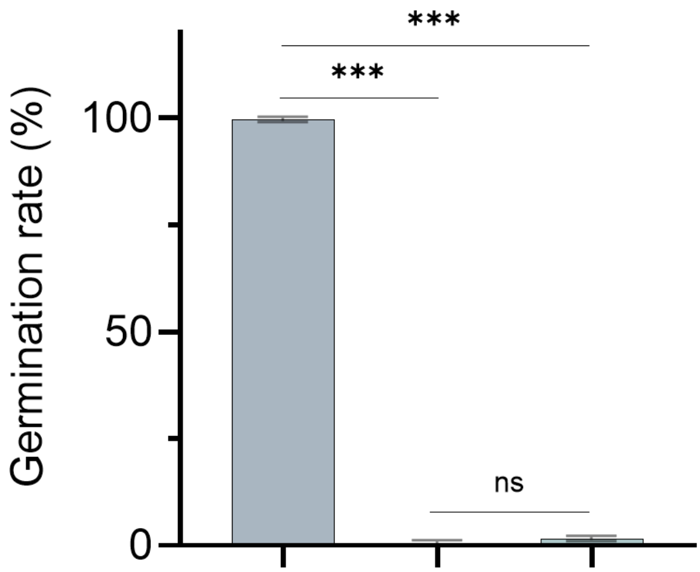

Fig. S3 Germination rate of rice seeds inoculated with ZJ-SD and ZJ-MD at a high concentration (1×10^8 CFU/mL). Values are means ± SD (shown as error bars) (n = 3). Statistical significance was determined by one-way ANOVA with Tukey's HSD test. (*$P < 0.05$, **$P < 0.01$, ***$P < 0.001$, ns, not significant).

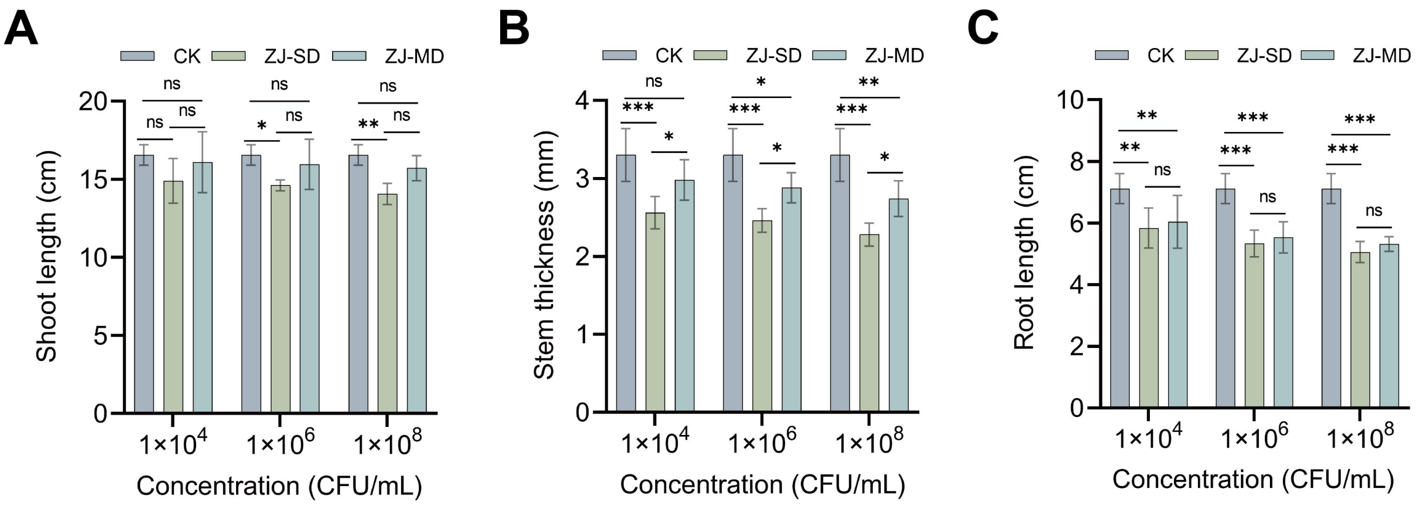

Fig. S4 Effects of ZJ-SD and ZJ-MD on rice growth in stem injection assays at the seedling stage. (**A-C**) Effects of ZJ-SD, ZJ-MD, and control (CK) on rice shoot length (A), stem thickness (B), and root length (C) after stem injection at the seedling stage with different bacterial concentrations (1×10^4, 1×10^6, and 1×10^8 CFU/mL). Values are means ± SD (shown as error bars) (n = 3). Statistical significance was determined by one-way ANOVA with Tukey's HSD test. (**P*< 0.05, ***P*<0.01, ****P*< 0.001, ns, not significant).

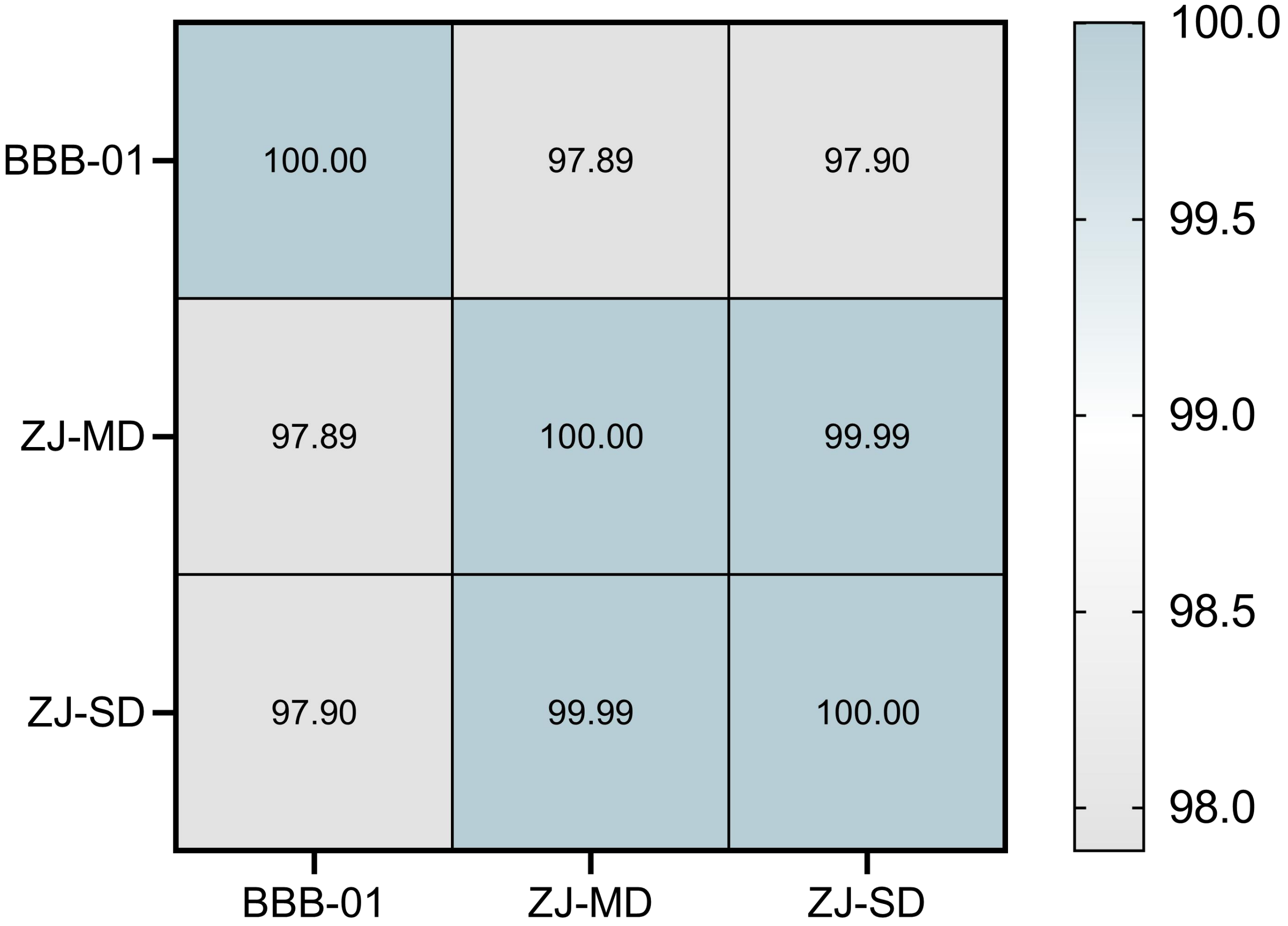

Fig. S5 Heatmap of pairwise average nucleotide identity (ANI) among strains ZJ-SD, ZJ-MD, and BBB-01. Numbers represent the percentage of nucleotide identity between genomes.

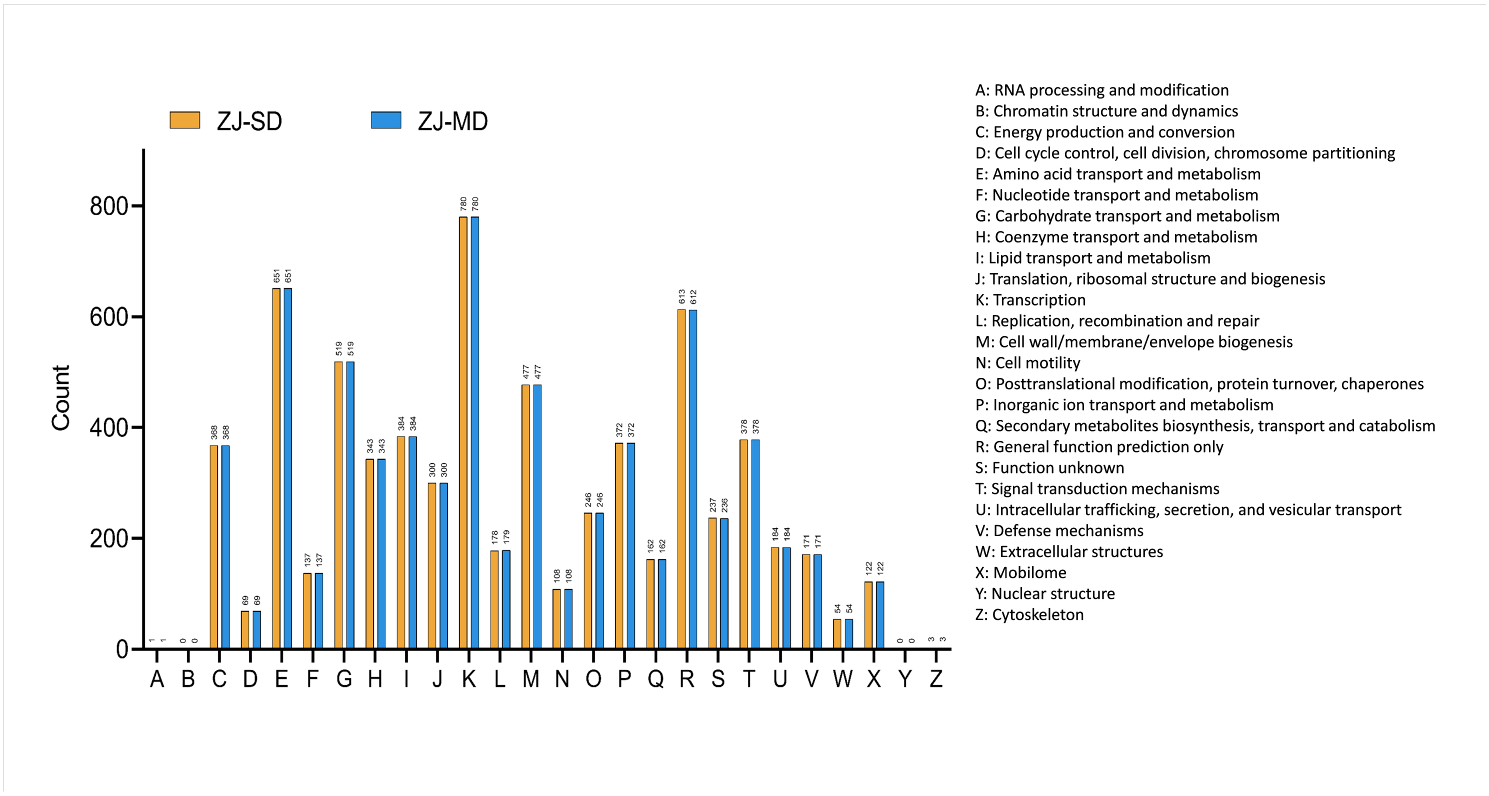

Fig. S6 COG functional classification of protein-coding genes in ZJ-SD and ZJ-MD. The number of protein-coding genes assigned to each COG functional category in ZJ-SD and ZJ-MD.

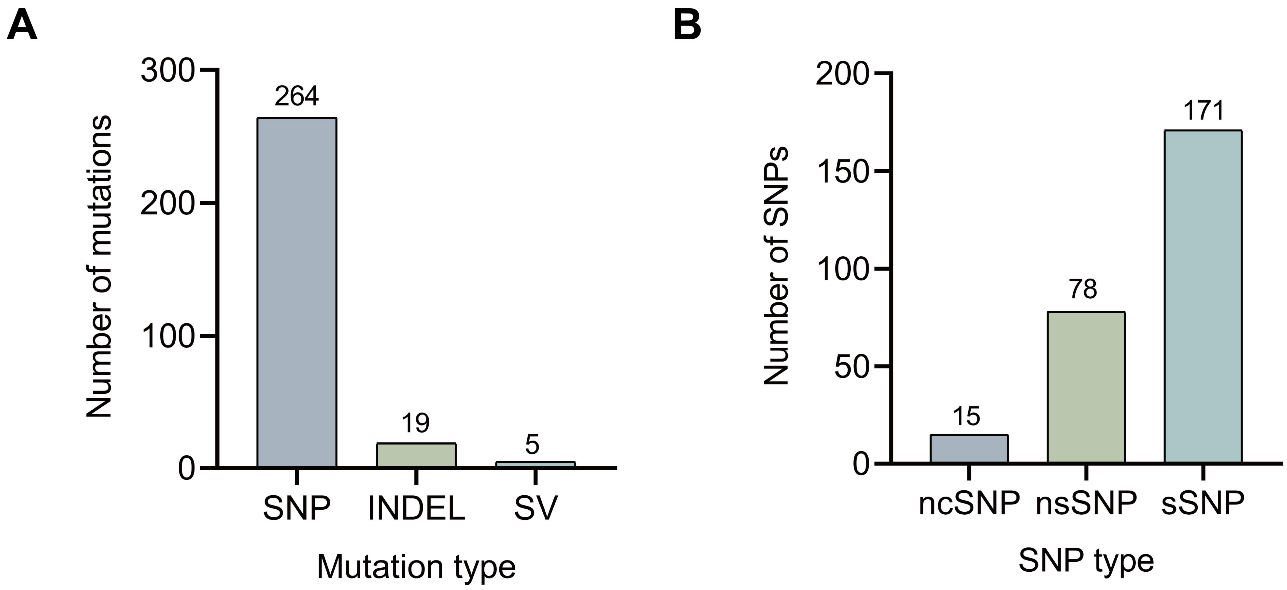

Fig. S7 Variation types and SNP classification in ZJ-MD compared to ZJ-SD. (**A**) Numbers of single nucleotide polymorphisms (SNPs), insertions and deletions (INDELs), and structural variations (SVs) identified in ZJ-MD compared to ZJ-SD. (**B**) Classification of SNPs as non-coding SNPs (ncSNPs), non-synonymous SNPs (nsSNPs), and synonymous SNPs (sSNPs).

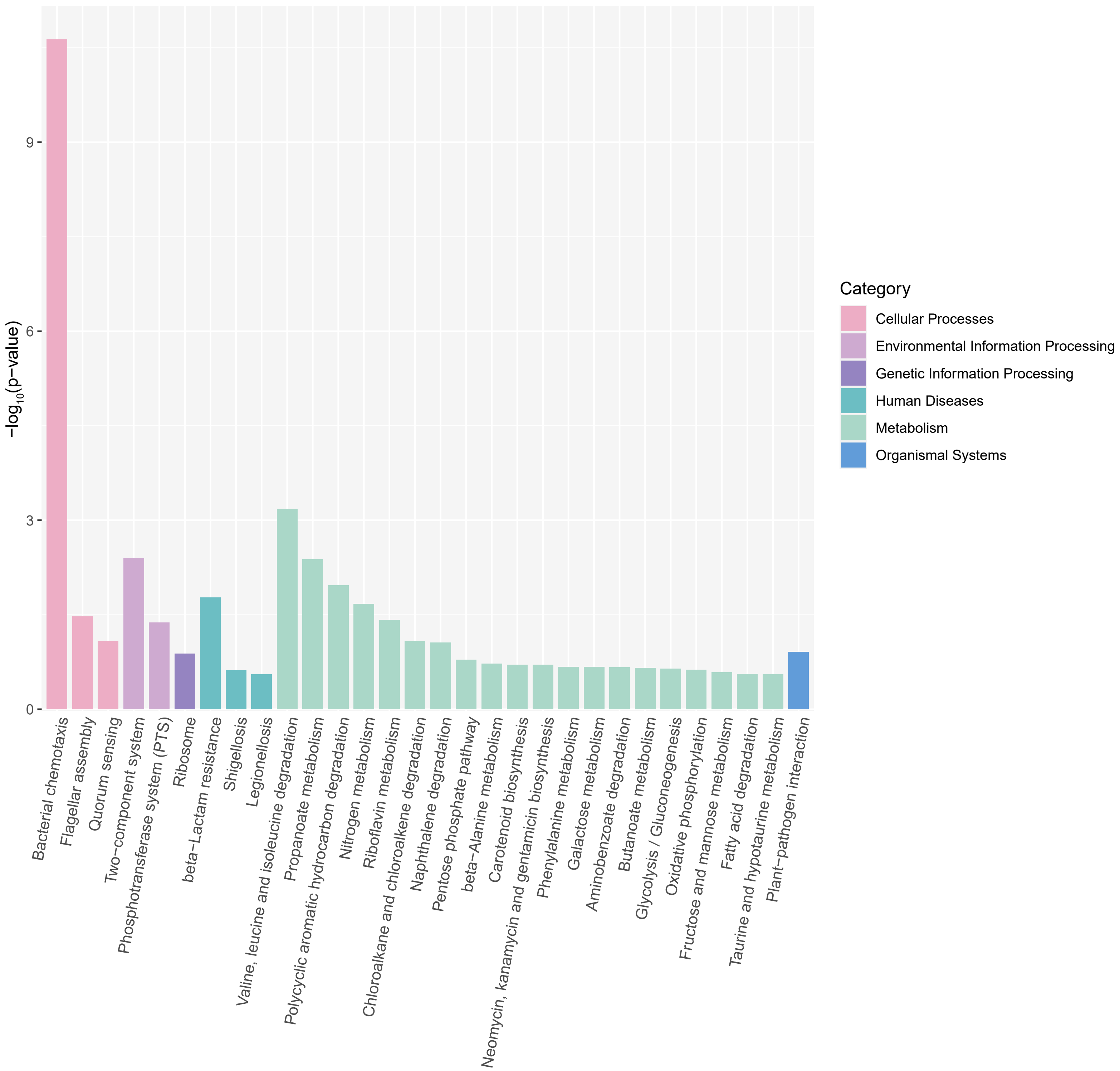

Fig. S8 KEGG pathway enrichment analysis of differentially expressed genes (DEGs). The top 20 significantly enriched KEGG pathways are shown, ranked by *p*-value. The x-axis indicates the pathway names, and the y-axis represents enrichment significance as -$\log_{10}$(*p*-value). Bar colors represent different KEGG pathway categories as indicated in the legend.
