# Supplementary material for: Multi-omics analyses reveal the virulence differentiation underlying natural variation in Burkholderia gladioli
Source: Appl Environ Microbiol. 2025 Nov 19;91(12):e01685-25. doi: 10.1128/aem.01685-25 (PMC12724388; doi:10.1128/aem.01685-25)
Supplement: Table S1 — Summary of 27 mutated genes and counts of SNPs, INDELs, and SVs. [file aem.01685-25-s0002.docx]

**Table S1** Summary of 27 mutated genes and counts of SNPs, INDELs, and SVs

| **Gene ID** | **Mutation Type** | **SNPs (count)** | **INDELs (count)** | **SVs (count)** |
| --- | --- | --- | --- | --- |
| BJLANFEE_00296 | nsSNPs | 1 | 0 | 0 |
| BJLANFEE_01313 | INDEL | 0 | 3 | 0 |
| BJLANFEE_01808 | nsSNPs | 1 | 0 | 0 |
| BJLANFEE_01821 | nsSNPs | 7 | 0 | 0 |
| BJLANFEE_01920 | nsSNPs | 6 | 0 | 0 |
| BJLANFEE_02684 | nsSNPs | 1 | 0 | 0 |
| BJLANFEE_02693 | nsSNPs | 1 | 0 | 0 |
| BJLANFEE_02717 | nsSNPs | 1 | 0 | 0 |
| BJLANFEE_02813 | nsSNPs | 6 | 0 | 0 |
| BJLANFEE_02814 | nsSNPs | 5 | 0 | 0 |
| BJLANFEE_03143 | nsSNPs | 1 | 0 | 0 |
| BJLANFEE_03943 | SV | 0 | 0 | 1 |
| BJLANFEE_04146 | nsSNPs | 1 | 0 | 0 |
| BJLANFEE_04393 | SV | 0 | 0 | 1 |
| BJLANFEE_05192 | nsSNPs | 4 | 0 | 0 |
| BJLANFEE_05428 | nsSNPs | 2 | 0 | 0 |
| BJLANFEE_05741 | nsSNPs, SV | 3 | 8 | 0 |
| BJLANFEE_05742 | nsSNPs | 4 | 0 | 0 |
| BJLANFEE_06188 | nsSNPs | 1 | 0 | 0 |
| BJLANFEE_06199 | INDEL | 0 | 1 | 0 |
| BJLANFEE_06233 | nsSNPs | 1 | 0 | 0 |
| BJLANFEE_06234 | nsSNPs | 1 | 0 | 0 |
| BJLANFEE_06726 | nsSNPs | 1 | 0 | 0 |
| BJLANFEE_06824 | nsSNPs | 27 | 0 | 0 |
| BJLANFEE_06958 | SV | 0 | 0 | 1 |
| BJLANFEE_07078 | nsSNPs | 1 | 0 | 0 |
| BJLANFEE_07080 | nsSNPs | 3 | 0 | 0 |
| Total |  | 79 | 12 | 3 |
